# A Complex Case of Unilateral Stasis Dermatitis With Id Reaction Misdiagnosed as Cellulitis: A Diagnostic Challenge

**DOI:** 10.7759/cureus.104555

**Published:** 2026-03-02

**Authors:** Sandra M Johnson, Youkyung S Roh, Sima Rozati

**Affiliations:** 1 Hospital Medicine, Johns Hopkins Health System, Baltimore, USA; 2 Dermatology, Johns Hopkins Health System, Baltimore, USA; 3 Dermatology, Johns Hopkins University School of Medicine, Baltimore, USA

**Keywords:** autoeczematization, dermatitis, id reaction, immune-mediated, stasis dermatitis

## Abstract

Autoeczematization, or id reaction, is a secondary eczematous eruption occurring at sites distant from a primary inflammatory skin process. It is often underrecognized in patients with stasis dermatitis and can mimic cellulitis or drug-induced eruptions. A male in his 50s with plaque psoriasis and a history of heavy smoking (55 pack years) presented with acute unilateral left leg erythema and edema, initially treated as recurrent cellulitis. He subsequently developed a diffuse, pruritic morbilliform eruption on the trunk and extremities. Biopsies revealed chronic spongiotic dermatitis in the leg and nonspecific spongiotic changes in the trunk. Imaging and cultures excluded infection or abscess. The eruption was diagnosed as autoeczematization secondary to stasis dermatitis. Topical corticosteroids, compression therapy, leg elevation, and skin care led to the gradual resolution of both local and widespread lesions. Clinicians should consider venous stasis in cases of unilateral leg swelling and recognize autoeczematization as a potential complication. Timely and accurate diagnosis can prevent unnecessary systemic therapies, with management focused on compression, topical corticosteroids, and supportive skin care.

## Introduction

Autoeczematization, also known as an id reaction, is a secondary, immune-mediated eczematous eruption that occurs at sites distant from a primary inflammatory skin process. It is classically a type IV hypersensitivity reaction and has been described in association with chronic dermatologic and infectious conditions, including stasis dermatitis, allergic contact dermatitis, and dermatophyte infections. Clinically, autoeczematization presents as a pruritic, erythematous, eczematous, and, occasionally, vesicular eruption that may mimic drug reactions, viral exanthems, or autoimmune disease.

Stasis dermatitis is a common inflammatory dermatosis of the lower extremities, typically associated with chronic venous insufficiency and characterized by erythema, scaling, edema, and pruritus. Atypical presentations, particularly unilateral disease, frequently lead to misdiagnosis as cellulitis and result in unnecessary antibiotic exposure. Unilateral stasis dermatitis may present even in the absence of overt signs of chronic venous insufficiency, such as leg discoloration, varicosities, or edema. A 2023 meta-analysis reported that more than one-third of patients hospitalized for presumed cellulitis were misdiagnosed, with stasis dermatitis representing the most common alternative diagnosis [1]. While stasis dermatitis typically presents bilaterally, the literature documents atypical unilateral presentations. For example, a 2009 study identified 37 cases presenting as solitary lesions, with the majority occurring in patients without prior clinical evidence of venous insufficiency [2].

Based on a comprehensive search of the literature, there are no recent published reports specifically quantifying cases of unilateral stasis dermatitis with secondary autoeczematization over the past five years. This absence may reflect the rarity of this presentation or underreporting in the medical literature. Recognizing atypical unilateral stasis dermatitis with secondary autoeczematization is clinically important to prevent misdiagnosis and unnecessary antibiotic use, particularly in cases initially suspected to represent cellulitis.

We present a case of acute unilateral leg erythema and edema complicated by a diffuse pruritic eruption, ultimately diagnosed as autoeczematization secondary to stasis dermatitis after initial misdiagnosis as cellulitis and drug-induced rash.

## Case presentation

A male in his 50s with a history of plaque psoriasis and a 55-pack-year smoking history presented with painful erythema and swelling of the left lower extremity, followed by the acute onset of a diffuse, intensely pruritic morbilliform rash involving the upper extremities, abdomen, and back. He was afebrile, hypertensive, with a heart rate of 60 beats/minute, and normal SpO₂ on room air (white blood cell count, 10.6 × 10³/µL; creatinine, 1.07 mg/dL; C-reactive protein, 1.4 mg/L; erythrocyte sedimentation rate, 26 mm/hour; blood and lower limb extremity bacterial, yeast, fungal, and mycobacterial cultures negative).

**Figure 1:**
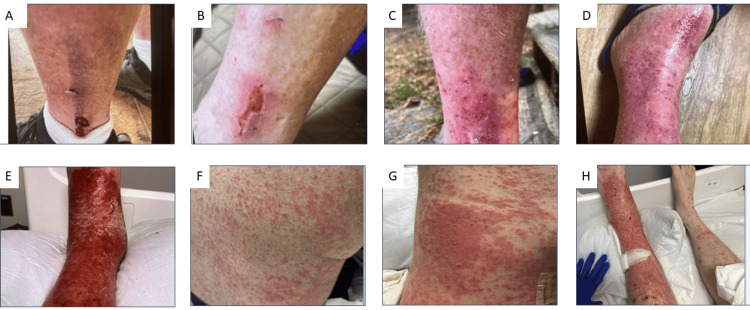
Timeline of progression of symptoms. Clinical progression of autoeczematization secondary to stasis dermatitis. A: Initial shin injury sustained in June. B: Healing of the initial injury following antibiotic treatment in September. C: Recurrent left leg erythema and edema in October. D: Recurrent leg erythema and edema after discontinuation of antibiotics and corticosteroids. E: Marked worsening of left leg erythema and edema at hospital presentation in November. F: Development of a widespread pruritic rash at the time of hospital presentation. G: Progression of the diffuse eruption over the following week. H: Persistent unilateral left leg erythema and edema exacerbated by dependent positioning.

The patient reported a five-month history of recurrent erythema and edema of the left lower leg following a superficial scrape sustained at a construction site. Given the unilateral presentation, absence of significant comorbidities, and history of localized trauma, he was initially diagnosed with cellulitis and treated with multiple courses of oral antibiotics and intermittent systemic corticosteroids at urgent care and emergency department visits. Symptoms temporarily improved during treatment courses but recurred following completion, with progressive worsening over time (Figures 1A-1D). Over subsequent weeks, the patient developed worsening left leg erythema and edema, accompanied by a progressive eruption that initially appeared morbilliform and later evolved into numerous erythematous, scaly papules and plaques involving the trunk and bilateral extremities. Lower extremity pain and swelling were exacerbated by prolonged standing and dependent positioning (Figures 1E-1H). Differential diagnoses at this stage included antibiotic-resistant cellulitis, rebound psoriasis following steroid withdrawal, scabies, bullous pemphigoid, and cutaneous lymphoma. He was admitted to the hospital and empirically started on intravenous antibiotics.

**Figure 2:**
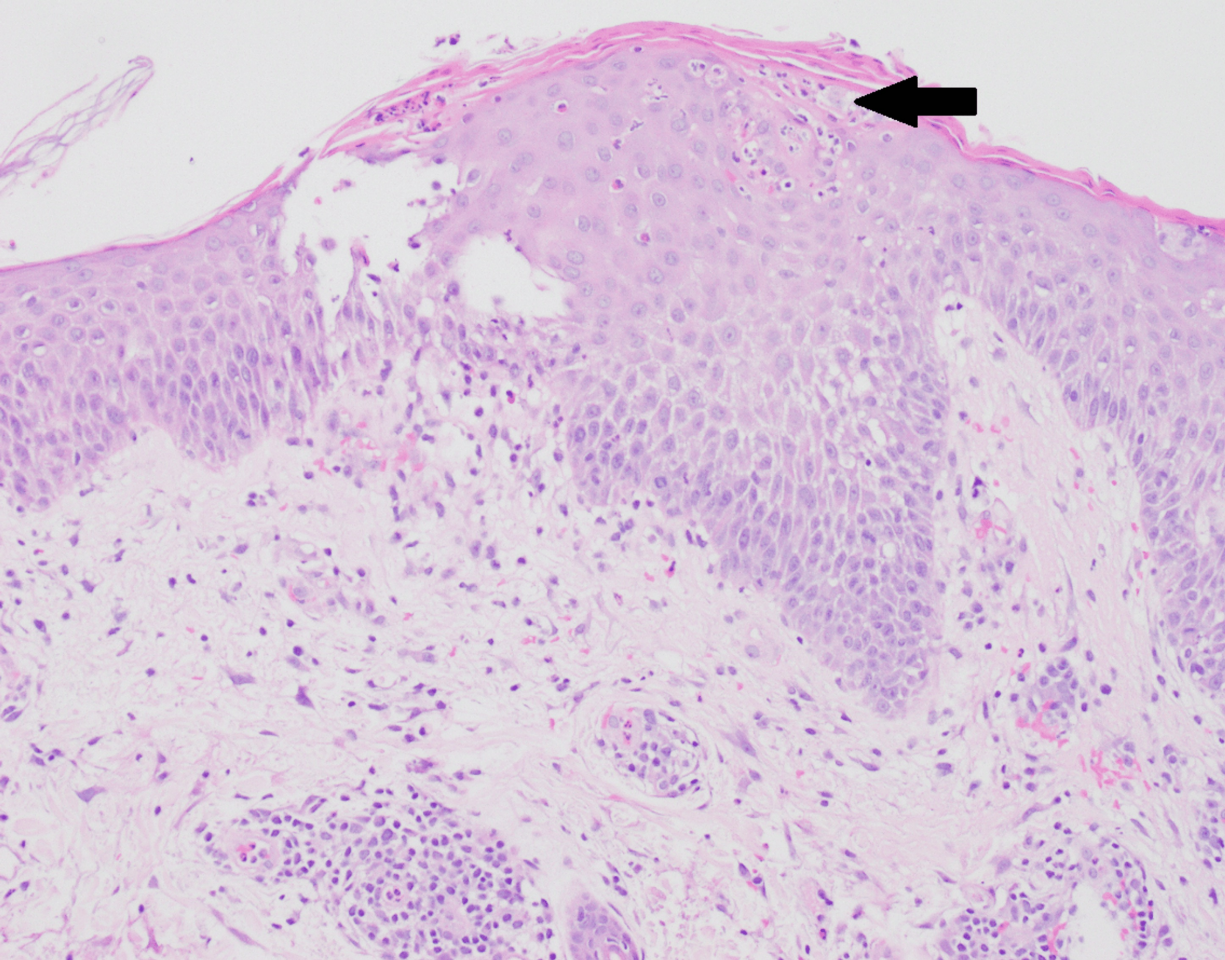
Right abdomen punch biopsy showing epidermal spongiosis and subcorneal pustules with eosinophils and neutrophils.

A punch biopsy of the abdominal rash demonstrated a nonspecific spongiotic dermatitis with subcorneal pustules, eosinophils, and neutrophils. Direct immunofluorescence was negative, favoring a possible drug-induced morbilliform eruption (Figure 2). The patient was started on topical triamcinolone and oral antihistamines.

**Figure 3:**
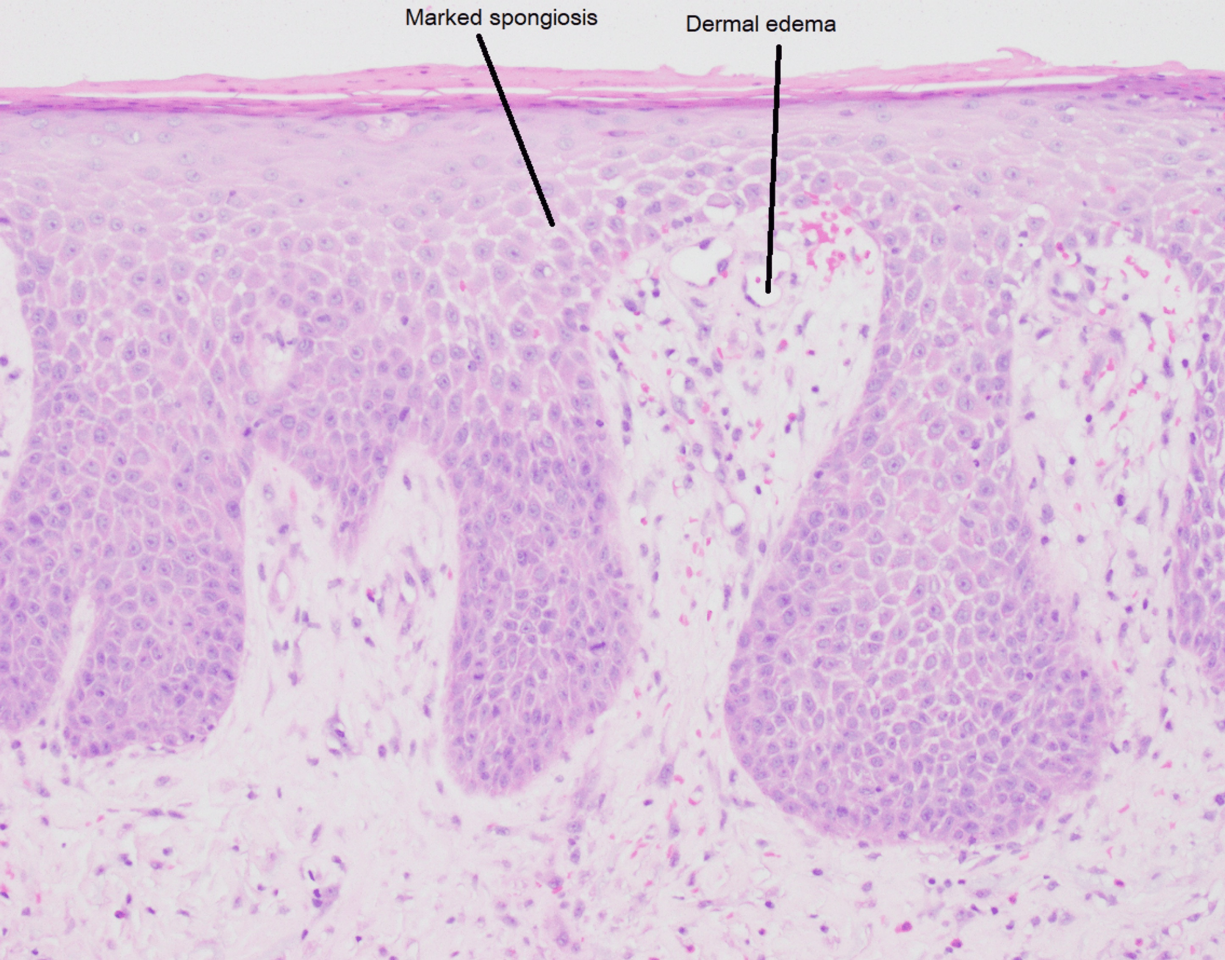
Left shin punch biopsy demonstrating chronic spongiotic dermatitis with eosinophils and dermal edema.

Tissue cultures from the left lower extremity were negative for bacterial, fungal, and mycobacterial organisms. Biopsy of the affected leg showed chronic spongiotic dermatitis with dermal edema and eosinophils, consistent with an eczematous process such as stasis dermatitis (Figure 3).

**Figure 4:**
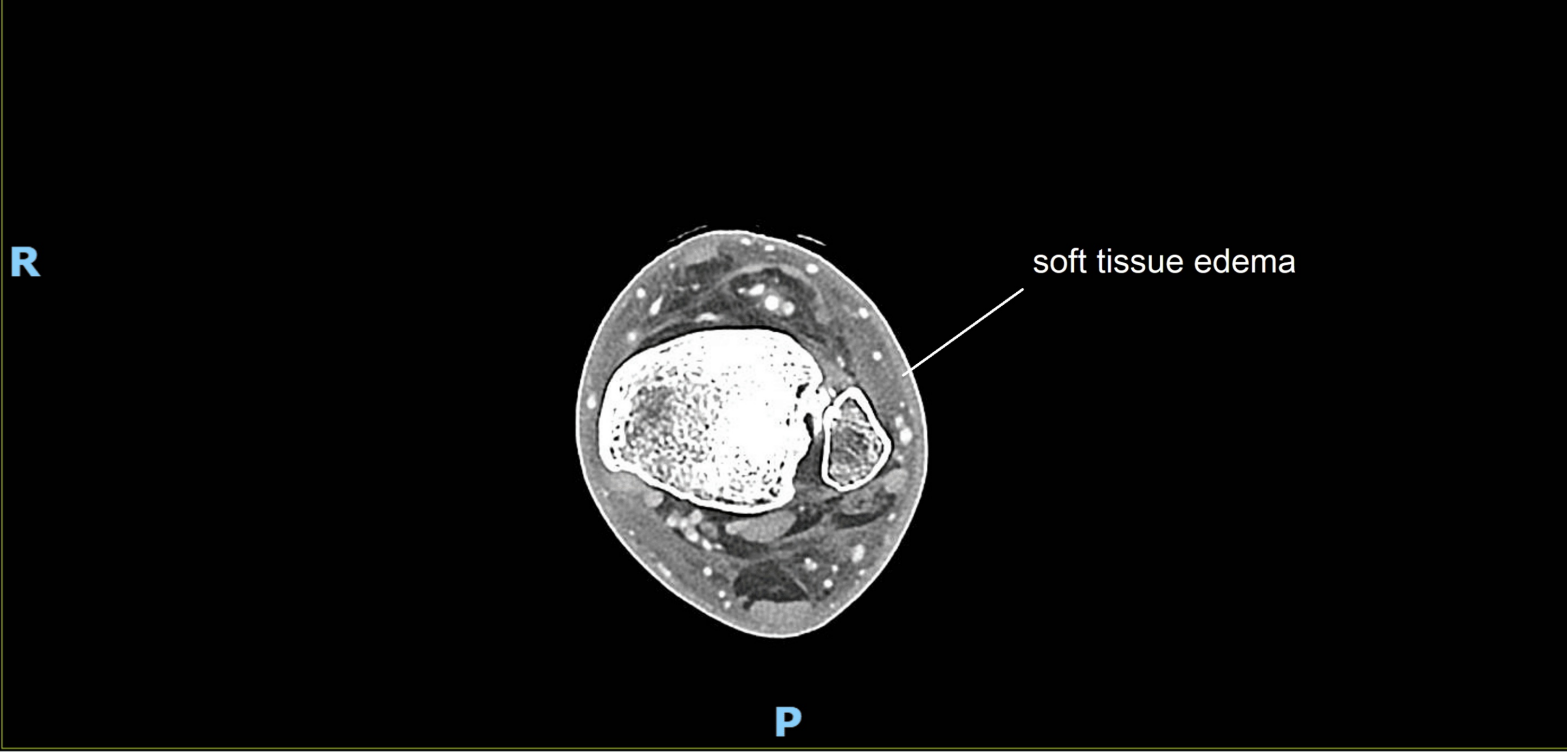
CT of the left lower extremity with intravenous contrast. Imaging demonstrating only soft-tissue edema, with no abscess or fluid collection or evidence of osteomyelitis.

Computed tomography of the left leg demonstrated diffuse soft tissue edema without abscess formation (Figure 4).

**Figure 5:**
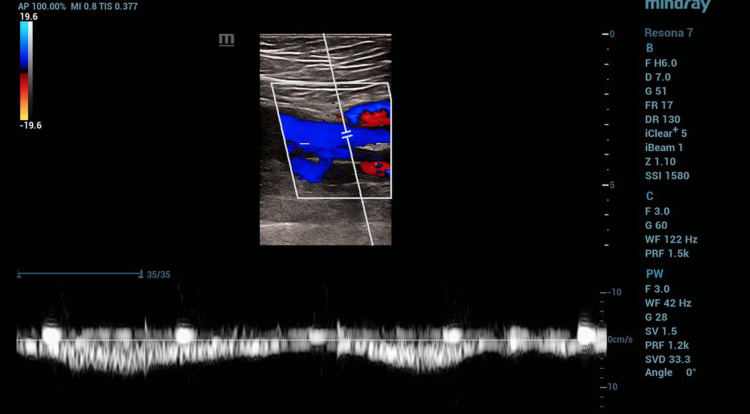
VAS venous reflux examination of the left side. Duplex evaluation demonstrated minimal reflux in the superficial venous system (GSV), with moderate reflux in the left femoral vein (deep venous system), but otherwise showed patent deep veins without thrombosis and no reflux in the greater or small saphenous veins.

Venous duplex and reflux studies revealed no deep vein thrombosis. Minimal superficial reflux at the saphenofemoral junction (1.5 seconds) was not clinically significant for surgical intervention, while moderate deep venous reflux in the left femoral vein (2.7 seconds) exceeded the one-second threshold for pathologic reflux (Figure 5). These findings support the diagnosis of stasis dermatitis due to superficial venous congestion, while acknowledging the presence of deep venous reflux that informs conservative management with compression therapy.

Based on the clinical course, as briefly summarized in Table 1, histopathologic findings, and exclusion of infectious and autoimmune etiologies, the diffuse truncal eruption was diagnosed as autoeczematization secondary to stasis dermatitis of the left lower extremity. The patient was treated with high-potency topical corticosteroids (clobetasol ointment) to the leg, compression stockings, leg elevation, and referral to a lymphedema specialist. The truncal eruption gradually resolved in parallel with the improvement of lower extremity edema.

**Table 1:** Clinical and therapeutic timeline of events. Timeline of key clinical events from initial LLE injury through post-discharge recovery. The table illustrates the temporal relationship between medications, rash onset, biopsy timing, and resolution, supporting distinction between drug eruption and autoeczematization. LLE: left lower extremity; HCTZ: hydrochlorothiazide; ED: emergency department

Date	Event/Intervention	Therapy/Medications	Key notes/Response	Category
7/29/2024	LLE ankle injury	—	Initially healed; later redness/swelling	LLE/Injury
8/22–9/13/24	LLE cellulitis	Keflex, doxycycline, augmentin, Bactrim (stopped)	Partial response; Bactrim stopped for rash	LLE/Infection
9/20/2024	ED: photosensitive rash	Hydroxyzine, Medrol pack	Rash attributed to doxycycline/HCTZ/valsartan/Bactrim	Rash/Drug reaction
9/30–10/5/24	Dermatology evaluation	Clobetasol, rriamcinolone; stop rosuvastatin and valsartan/HCTZ	Hand rash improved; LLE erythema persists	Rash/Drug reaction and LLE
10/5–10/23/24	Persistent LLE symptoms	Supportive care; ibuprofen and antihistamines	LLE swelling/erythema continued	LLE/Infection
10/23/2024	Morbilliform rash on the back	—	Patient stopped amlodipine and clonidine	Rash/Drug reaction
10/25/2024	Hospitalization	Vancomycin	LLE cellulitis; inpatient monitoring	LLE/Infection
10/27–10/28/24	Rash worsening; biopsy	—	Abdominal rash biopsy performed	Rash/Drug reaction
10/30/2024	LLE biopsy results	—	Cultures negative; eczematous process confirmed	LLE/Non-infectious
11/4/2024	Post-discharge	Topical steroids, LLE elevation and compression	LLE pain/swelling resolved; mild lymphedema persists	LLE/Recovery
12/13/2024	Rash resolution	—	Complete resolution	Rash/Drug reaction
Ongoing	Outpatient follow-up	Compression stockings; lymphedema clinic; vascular surgery	Monitor for recurrence or complications	LLE/Recovery

## Discussion

This case highlights the diagnostic challenges of stasis dermatitis when autoeczematization obscures the underlying pathology and classic features of chronic venous insufficiency are absent. The patient initially presented with localized erythema, warmth, and tenderness, making cellulitis the leading diagnostic consideration. However, the absence of fever, systemic symptoms, and elevated inflammatory markers, together with negative blood and tissue cultures and poor response to multiple antibiotic courses, argued strongly against an infectious etiology [1].

Given the patient’s history of plaque psoriasis and recent steroid tapers, a rebound psoriasis flare was considered but was unsupported by the acute onset, atypical distribution, and nonspecific histopathologic findings. Other potential diagnoses, including venous and arterial ulcers, vasculitis, pretibial myxedema, lichen simplex chronicus, and lymphedema, were explored, yet the clinical, serologic, and histologic evaluations did not align with these conditions [2-4]. A summary of key differential diagnoses and distinguishing clinical features is provided in Table 2. As the rash progressed into a diffuse, pruritic eruption involving the trunk and extremities, drug-induced reactions, viral exanthems, atopic dermatitis, and contact dermatitis were considered; however, the temporal course, distribution, and lack of systemic findings or relevant exposures made these diagnoses less plausible [2-5].

**Table 2:** Differential diagnoses of stasis dermatitis. Conditions with overlapping clinical features were considered in the differential diagnosis of unilateral lower extremity erythema and edema. Key distinguishing factors, diagnostic tests, and treatments are summarized. Legend independently created by the authors using information from the cited sources [3-5].

Condition	Key features	Distinguishing factors	Diagnostic tests	Treatment
Stasis dermatitis	Erythema, swelling, pruritic rash, usually in the lower legs	Associated with venous insufficiency, chronic leg swelling	Clinical exam, venous duplex ultrasound	Compression therapy, topical steroids, moisturizers
Cellulitis	Rubor, warm, swollen skin, pain, often with fever	Rapid onset, well-defined borders, systemic symptoms including fever	Clinical exam, blood cultures, wound cultures	Oral or IV antibiotics, drainage if abscess present
Contact dermatitis	Erythematous, itchy rash, often in areas of skin contact	History of exposure to irritants or allergens	Patch testing, clinical exam	Avoidance of irritants, topical steroids, antihistamines
Psoriasis	Well-demarcated, scaly plaques, often with silvery scales	Chronic condition with remissions; typically on scalp, elbows, knees	Clinical exam, skin biopsy (if needed)	Topical corticosteroids, vitamin D analogs, phototherapy
Venous ulcers	Chronic leg ulcers with surrounding erythema and edema	Medial lower leg ulcers, associated with varicose veins and venous insufficiency	Clinical exam, Doppler ultrasound	Compression therapy, wound care, surgical intervention as needed
Arterial ulcers	Painful ulcers on distal extremities (toes, heels); cool, pale skin	History of peripheral arterial disease; diminished pulses	Ankle-brachial index, arterial Doppler, angiography	Revascularization, wound care, antiplatelet therapy
Leukocytoclastic vasculitis	Palpable purpura, especially on lower legs; may ulcerate	Immune complex-mediated; often post-infectious or drug-related	Skin biopsy, urinalysis, antineutrophil cytoplasmic antibodies, complement levels	Treat underlying cause, systemic steroids, immunosuppressants
Pretibial myxedema	Firm, non-pitting, waxy plaques on shins; often bilateral	Associated with Graves’ disease (thyroid dermopathy)	Thyroid-stimulating hormone, T3, T4, thyroid antibodies	Treat underlying thyroid disease, topical steroids, compression wraps
Lymphedema	Chronic non-pitting swelling, usually in one or both lower extremities	Often painless; thickened skin (peau d’orange); recurrent infections	Lymphoscintigraphy, clinical exam	Compression therapy, manual lymphatic drainage, skin care
Lichen simplex chronicus	Thickened, scaly plaques caused by chronic scratching or rubbing	Localized; intense pruritus; history of repeated irritation	Clinical diagnosis; skin biopsy if uncertain	Stop scratching, high-potency topical steroids, antihistamines
Necrobiosis lipoidica	Yellow-brown atrophic plaques, often on shins; may ulcerate	Strongly associated with diabetes mellitus	Clinical exam, skin biopsy	Variable: topical corticosteroids, phototherapy, biologics, or JAK inhibitors

Biopsies of the left leg and truncal lesions demonstrated chronic spongiotic dermatitis with dermal edema, consistent with stasis dermatitis complicated by secondary autoeczematization (Figures 2, 3). Although vascular studies, including duplex ultrasound and ankle-brachial index testing, were largely unremarkable, clinical features such as unilateral dependent edema worsened by standing, history of local trauma, and chronic smoking supported localized venous dysfunction as the underlying driver. Notably, the patient had no prior history or classic cutaneous signs of chronic venous insufficiency, such as hyperpigmentation, varicosities, or lipodermatosclerosis, which made the diagnosis less clinically apparent. We postulate that the inciting leg injury, in combination with chronic tobacco use and prolonged occupational standing, contributed to subclinical venous hypertension that became clinically evident only after trauma. This atypical presentation underscores the importance of considering venous stasis even in the absence of established chronic skin changes, particularly in cases of presumed cellulitis that fail to respond to appropriate antibiotic therapy.

The abdominal rash showed nonspecific spongiotic dermatitis with subcorneal pustules, eosinophils, and neutrophils; direct immunofluorescence was negative, suggesting a possible drug-induced morbilliform eruption. While histopathology supported an eczematous process, it was not specific in isolation. The final diagnosis of stasis dermatitis with autoeczematization was established through clinicopathologic correlation, consideration of the clinical course, integration of imaging and vascular studies, and exclusion of infectious etiologies.

Autoeczematization is characterized by the sudden dissemination of a previously localized eczematous process to distant skin sites, resulting in widespread secondary eczematous lesions. It is most commonly associated with chronic localized dermatoses, particularly stasis dermatitis, but has also been reported in occupational contact dermatitis and as an adverse effect of certain dermatologic therapies [6,7]. In the setting of stasis dermatitis, autoeczematization typically manifests as pruritic eruptions distant from the lower extremities and may assume a nummular morphology, mimicking primary eczema, drug eruptions, viral exanthems, or even cutaneous lymphoma. These overlapping clinical features frequently contribute to misdiagnosis and inappropriate treatment. Common conditions that may mimic autoeczematization and their distinguishing characteristics are outlined in Table 3 [5-12].

**Table 3:** Differential diagnoses of id reactions. Autoeczematization (id reaction) can mimic various dermatologic conditions. Key features and distinguishing characteristics of commonly considered alternatives are summarized. Legend independently created by the authors using information from the cited sources [6-18].

Condition	Key features	Distinguishing features
Contact dermatitis	Erythema, pruritus, scaling, vesicles	Occurs at the site of contact with allergen/irritant, well-defined borders and geometric shapes
Eczema (atopic dermatitis)	Chronic, pruritic, erythema, scaling, often with lichenification	Typically, in flexural areas, a personal/family history of atopy
Psoriasis	Well-demarcated, silvery-scaly plaques, often on extensor surfaces	Thick, silvery scales, family history
Tinea (fungal infections)	Annular, raised plaques with scaly edges and central clearing	Characteristic annular lesions with a well-defined border
Viral exanthems	Often non-specific morbilliform rash, can be associated with systemic symptoms (fevers, lymphadenopathy, etc.)	Specific patterns and distribution related to viruses
Drug eruptions	Variable; maculopapular, urticarial, or vesicular rash	Linked to a new medication, resolves with discontinuation
Scabies	Intense pruritus, small erythematous papules, burrows in webbed areas	Burrows, typically localized to specific areas, not generalized
Vasculitis	Inflammatory skin changes, purpura, petechiae, ulcerations	Palpable purpura, systemic symptoms, autoimmune associations
Herpes simplex virus	Painful, grouped vesicles on an erythematous base	Localized lesions, recurrent outbreaks
Urticarial bullous pemphigoid	Tense blisters on a urticarial or eczematous base, intense pruritus	Subepidermal blisters, positive BP180/BP230 antibodies, elderly patients, responds to immunosuppressants
Cutaneous lymphoma	Persistent patches, plaques, or nodules, often scaly or ulcerated	Chronic, slowly progressive; biopsy shows atypical lymphocytes; may mimic eczema or psoriasis

The pathophysiology of autoeczematization in stasis dermatitis remains incompletely understood but appears to reflect immune-mediated amplification of inflammation rather than direct spread of infectious or antigenic material. Chronic venous insufficiency promotes sustained local inflammation through venous hypertension, leukocyte trapping, endothelial activation, and red blood cell extravasation. Hemosiderin deposition contributes to ongoing tissue injury and metalloproteinase upregulation, while macrophage activation and pruritogenic cytokines, particularly interleukin-31, play a central role in propagating inflammation and pruritus. A Th2-skewed immune response, mediated by cytokines such as IL-4, IL-13, and IL-31, further drives epidermal barrier dysfunction and dissemination of eczematous inflammation beyond the primary lesion. Together, these mechanisms help explain how a localized venous inflammatory process can manifest as a generalized dermatologic eruption [12-18].

Clinically, autoeczematization is distinguished from primary eczema by evidence of underlying venous disease and a temporal pattern of sudden dissemination from a localized focus [8,9]. Failure to recognize this entity often leads to repeated antibiotic exposure or systemic corticosteroid use, which may provide transient improvement but fail to address the underlying inflammatory driver and can precipitate rebound flares. In this case, repeated misdiagnosis as cellulitis resulted in multiple courses of antibiotics and intermittent systemic corticosteroids, with only temporary symptom relief [5]. Negative cultures, absence of systemic infection, and histopathologic findings ultimately clarified the diagnosis. To enhance practical applicability, a focused comparison of distinguishing features between cellulitis and stasis dermatitis with secondary autoeczematization is provided in Table 4.

**Table 4:** Clinical features distinguishing cellulitis from stasis dermatitis with autoeczematization. Practical comparison of cellulitis and stasis dermatitis with secondary autoeczematization. The table summarizes distinguishing clinical features, laboratory findings, imaging characteristics, and therapeutic response patterns to aid clinicians in differentiating infectious from inflammatory etiologies of unilateral lower extremity erythema. Legend independently created by the authors using information from the cited sources [1-6].

Feature	Cellulitis	Stasis dermatitis ± autoeczematization
Onset	Acute, progressive over hours to days	Subacute or chronic; may flare after trauma
Laterality	Usually unilateral	Often bilateral but may be unilateral
Pain vs. pruritus	Pain, tenderness prominent	Pruritus more prominent than pain
Systemic signs	Fever, chills common	Typically afebrile
Laboratory findings	Leukocytosis, elevated C-reactive protein/erythrocyte sedimentation rate common	Often normal inflammatory markers
Response to antibiotics	Clinical improvement within 48–72 hours	Minimal or no improvement
Edema pattern	Localized inflammatory swelling	Dependent edema worsened by standing
Distant rash	Uncommon	Possible secondary id reaction (diffuse eczematous eruption)
Imaging	Soft tissue edema ± abscess	Venous insufficiency or reflux may be present

For this patient, management directed at the underlying venous dysfunction, through compression therapy, topical corticosteroids, and supportive skin care, led to significant improvement in both localized and disseminated disease [16,18]. Clinical improvement was noted within approximately 7-10 days of initiating therapy, with marked reduction in pain and erythema, and substantial improvement in lower extremity swelling, although mild residual lymphedema persisted. The diffuse truncal eruption gradually resolved over four to six weeks, with complete resolution documented by 12/13/2024. Lifestyle modifications, including leg elevation, smoking cessation, and appropriate skin care, further contributed to symptomatic improvement. At ongoing outpatient follow-up, there has been no recurrence of diffuse autoeczematization, though mild dependent swelling continues intermittently with prolonged standing.

This case underscores the importance of considering venous stasis in the differential diagnosis of unilateral leg swelling and erythema, recognizing autoeczematization as a potential complicating feature, and prioritizing targeted therapy that addresses the primary vascular and inflammatory processes rather than defaulting to systemic antibiotics or corticosteroids.

## Conclusions

This case highlights the diagnostic challenges that may arise with atypical presentations of stasis dermatitis complicated by autoeczematization. Acute unilateral leg erythema followed by a diffuse pruritic dermatitis can mimic cellulitis, drug reactions, or autoimmune conditions, potentially leading to unnecessary antibiotic or systemic corticosteroid exposure. Although conclusions are limited by the nature of a single case, our experience suggests that earlier recognition of stasis dermatitis and associated id reactions may help reduce misdiagnosis and facilitate more targeted management. Clinicians may consider autoeczematization in the differential diagnosis when a widespread eczematous eruption develops in the setting of localized inflammatory or vascular findings, particularly when presumed cellulitis does not respond to appropriate therapy. This case further supports the potential value of multidisciplinary evaluation and careful reassessment of non-resolving unilateral leg eruptions.
